# *Mycobacterium tuberculosis* Rv0927c Inhibits NF-κB Pathway by Downregulating the Phosphorylation Level of IκBα and Enhances Mycobacterial Survival

**DOI:** 10.3389/fimmu.2021.721370

**Published:** 2021-08-31

**Authors:** Aihong Xia, Xin Li, Juanjuan Quan, Xiang Chen, Zhengzhong Xu, Xinan Jiao

**Affiliations:** ^1^Jiangsu Key Laboratory of Zoonosis/Jiangsu Co-Innovation Center for Prevention and Control of Important Animal Infectious Diseases and Zoonoses, Yangzhou University, Yangzhou, China; ^2^Key Laboratory of Prevention and Control of Biological Hazard Factors (Animal Origin) for Agrifood Safety and Quality, Ministry of Agriculture and Rural Affairs, Yangzhou University, Yangzhou, China

**Keywords:** Rv0927c, *Mycobacterium tuberculosis*, proinflammatory cytokine, NF-κB pathway, mycobacterial survival

## Abstract

Through long-term coevolution with its host, *Mycobacterium tuberculosis* (*M. tuberculosis*) uses multiple strategies to escape host defenses. The *M. tuberculosis* Rv0927c protein is predicted to be a short-chain dehydrogenase/reductase related to bacterial metabolism. However, the role of Rv0927c during *M. tuberculosis* infection remains unclear. Here, we observed that Rv0927c inhibited the expression of IL-6, TNF-α, and IL-1β, an effect dependent on NF-κB and p38 pathways. Western blot analysis of macrophages infected with recombinant *Mycobacterium smegmatis* strains showed that Rv0927c attenuated NF-κB activation by downregulating the phosphorylation of IκBα. Additionally, Rv0927c enhanced intracellular survival of *M. smegmatis* and pathological effects in mice. In conclusion, our findings demonstrate that Rv0927c functions as a regulator of inflammatory genes and enhances the survival of *M. smegmatis*.

## Introduction

Tuberculosis (TB) is a communicable disease caused by *Mycobacterium tuberculosis* (*M. tuberculosis*) complex (1). TB led to an estimated 1.2 million deaths among HIV-negative people in 2019 and an additional 208,000 deaths among HIV-positive people (2). Macrophages are the first line of defense in hosts. However, their failure to eliminate the bacteria completely may lead to mycobacteria replication in host cells (3, 4). Therefore, understanding the interactions between the pathogen and host cells is essential to find more effective strategies for controlling mycobacterial infection.

The initiation of host immunity is based on recognizing conserved molecular patterns of pathogens, which trigger signaling through Toll-like receptors (TLRs) (5). When ligands bind to TLRs, the receptors immediately recruit MyD88 from the cytoplasm to the cell membrane. Assisted by the bridging protein Mal, MyD88 is linked through the TIR domain and recruits IRAK-4 to the receptor complex (6). Upon binding IRAK-4 to the receptor complex through MyD88, the preformed Tollip/IRAK-1 complex in the cytoplasm is recruited to the activated receptor complex. IRAK-1 binds to MyD88 through its death domain and dissociates from Tollip (7). Activated IRAK-4 induces the autophosphorylation of IRAK-1 and the phosphorylated IRAK-1 rapidly binds to TRAF6, allowing TRAF6 to be recruited into the receptor complex. Subsequently, the TRAF6-IRAK-1 complex dissociates from the receptor complex and interacts with the preformed TAK1-TAB2-TAB3 complex on the cell membrane. Mediated by phosphorylated IRAK-1, the complex is translocated from the cell membrane to the cytoplasm and interacts with Ubc13 and Uev1A to form Lys63-linked polyubiquitination chains (8). The zinc finger domain of TAB2 or TAB3 recognizes the Lys63-linked polyubiquitination chains to form a complex that facilitates the ubiquitination and phosphorylation of TAK1 (9). Finally, activated TAK1 triggers IKK and MAPKs. Degradation of IκBα proteins leads to the subsequent translocation of the nuclear factor kappa B (NF-κB) and activation of the MAPK pathways leads to the induction of the transcription factor AP-1 (10), which regulate the expression of proinflammatory cytokines. Inactivation of NF-κB or MAPK pathways has been reported to decrease the expression of proinflammatory cytokines, but increase bacterial burden and histopathological changes in *M. tuberculosis* infected mice (11–13).

The production of proinflammatory cytokines is essential for controlling *M. tuberculosis* infection (1, 14). Unfortunately, *M. tuberculosis* can suppress the expression of proinflammatory cytokines by interfering with a range of processes in NF-κB and MAPKs pathways. Rv0222 utilizes E3 ubiquitin ligase anaphase promoting complex (APC) subunit 2 (ANAPC2) to induce K11-linked ubiquitination. K11-linked ubiquitination of Rv0222 promotes the recruitment of protein tyrosine phosphatase SHP1 to TRAF6, which prevents TRAF6 activation, followed by the inhibition of proinflammatory cytokine gene expression (15). In addition, PtpA can act as a phosphatase to directly dephosphorylate p-Jnk and p-p38 proteins in host cells, thereby blocking the activation of MAPK signaling pathways. In addition, PtpA competes with tab3, a key signal transduction molecule in the NF-κB signaling pathway, to bind the ubiquitin chain, thereby inhibiting the NF-κB signaling pathway (16). Furthermore, Mce3E can competitively bind ERK1/2 with MEK1 through its DEF motif (a motif that specifically binds ERK1/2 and other MAPK molecules), thereby inhibiting the phosphorylation of ERK1/2. Moreover, Mce3E inhibits the localization of p-ERK1/2 in the nucleus by trapping it in the endoplasmic reticulum (17). These proteins allow *M. tuberculosis* to evade immune surveillance and promote chronic infections.

The *M. tuberculosis* gene *Rv0927c* is 792 bp long and is present in *M. tuberculosis* complex group and *Mycobacterium bovis* attenuated strain BCG. *Rv0927c* may encode a short dehydrogenase/reductase and is related to the synthesis of mycotic acid in the cell wall of mycobacteria (18). *M. tuberculosis* transposition mutant Tn : *Rv0927c* induced more TNF-α, IL-6, COX-2, and iNOS expression, when compared with wild-type *M. tuberculosis* (19), suggesting that Rv0927c may act as an immune regulator to alter the fate of intracellular mycobacteria. However, a detailed understanding of its role in mycobacterial infection is currently lacking. Our data show that Rv0927c inhibited proinflammatory cytokine production *in vivo* and *in vitro* and enhanced mycobacterial survival.

## Materials and Methods

### Cell Line and Bacterial Cultures

RAW264.7 cells (ATCC, Manassas, VA, USA) were cultured in complete DMEM (Gibco, Grand Island, NY, USA) containing 10% Fetal Bovine Serum (FBS, Gibco), 100 U/ml streptomycin and 100 U/μl penicillin (Gibco) at 37°C and 5% CO_2_. Primary bone marrow-derived macrophages (BMDMs) were obtained from C57BL/6 mouse bone marrow as described previously (20). BMDMs were cultured in complete DMEM supplemented with 25 ng/ml macrophage colony-stimulating factor (M-CSF, PeproTech, RockyHill, NJ, USA) for 3–6 days. HEK293-TLR4 cells (InvivoGen, San Diego, CA, USA) were cultured in complete DMEM supplemented with 100 μg/ml Normocin™ (InvivoGen) and 10 μg/ml Blasticidin (InvivoGen). *Escherichia coli* DH5α (TaKaRa, Dalian, China) was cultured in LB medium for DNA cloning. *M. smegmatis* strain mc^2^ 155 (Our laboratory) and recombinant *M. smegmatis* were cultured in Middlebrook 7H9 (7H9, BD Biosciences, Franklin Lakes, NJ, USA) liquid medium containing 0.05% (v/v) Tween 80 with shaking or on Middlebrook 7H10 (7H10, BD) agar supplemented with 0.5% (v/v) glycerol.

### Animals and Ethics Statement

C57BL/6 mice were obtained from the Comparative Medical Center of Yangzhou University (Yangzhou, China) and kept in specific pathogen-free conditions, in the mouse isolators (Suzhou monkey animal experiment equipment Technology, Suzhou, China). All animal experiments were approved by the Animal Welfare and Ethics Committees of Yangzhou University and complied with the guidelines of the Institutional Administrative Committee and Ethics Committee of Laboratory Animals (IACUC license number: YZUDWLL-201811-001). Six to eight weeks old C57BL/6 mice were used for BMDMs preparation. Six weeks old C57BL/6 mice were intraperitoneally infected with 5×10^7^ CFU of recombinant *M. smegmatis* strains per animal (five mice per group). Spleens, livers and lungs of mouse were collected after infection at 3 days or 6 days. Tissues were weighted and homogenized for CFU analysis. The samples of mouse livers were fixed in 10% neutral formalin fix solution and stained with hematoxylin and eosin (H&E) for evaluation of pathologic changes.

### Construction of Recombinant Plasmids and *M. smegmatis* Strains

The full-length of *Rv0927c* gene was amplified from the genomic DNA of *M. tb* H37Rv by PCR using appropriate primers listed in **Table 1**. PCR product was cloned into pMV261 or pCMV to generate pMV261-*Rv0927c* and pCMV-*Rv0927c*. Then, the recombinant plasmid pMV261-*Rv0927c* was electroporated into *M. smegmatis* mc^2^155, while pCMV-*Rv0927c* was transformed into *Escherichia coli* (*E. coli*) DH5α cells by heat shock according standard procure. Expression of Rv0927c was confirmed by immunoblotting.

**Table 1:** Primers used in this study.

Primer Name	Primer Sequence
pMV261-*Rv0927c*-F	TCGGATCCATGATCCTGGATATGTTC (*Bam*H I)
pMV261-*Rv0927c*-R	TAGAATTCTCA*GTGATGATGGTGATGATG*CAGGTCCGGAATGGGAA (*Eco*R I; *His Tag*)
pCMV-*Rv0927c*-F	TGGCCATGGAGGCCCGAATTCGGATGATCCTGGATATGTTCCGTC (*Eco*R I)
pCMV-*Rv0927c*-R	CCGCGGCCGCGGTACCTCGAGTCACAGGTCCGGAATGGGAA (*Xho* I)
M-*IL-6*-F	TACCACTCCCAACAGACC
M-*IL-6*-R	CATTTCCACGATTTCCCAGA
M-*TNF-α*-F	TCTCATTCCTGCTTGTGG
M-*TNF-α*-R	ACTTGGTGGTTTGCTACGA
M-*IL-1β*-F	GCCACCTTTTGACAGTGATG
M-*IL-1β*-R	TGATGTGCTGCTGCGAGA
M-*GAPDH*-F	CAAATTCAACGGCACAGTCA
M-*GAPDH*-R	TTAGTGGGGTCTCGCTCC

### Preparation of Rv0927c Protein

*Rv0927c* gene was synthesized and ligated into pET30a(+) expression vector (DetaiBio, Nanjing, China). Recombinant plasmid was transformed into *E. coli* BL21(DE3) cells and transformed *E. coli* cells were induced to express recombinant protein. The rHis-Rv0927c protein was purified with a His-binding purification kit (Novagen, Madison, WI). Endotoxins were removed from Rv0927c protein by ToxinEraser™ (GenScript, Nanjing, China). Endotoxin level of Rv0927c was <0.05 EU/μg determined by ToxinSensor™ (GenScript).

### Growth Curves of Recombinant *M. smegmatis* and Subcellular Location of Rv0927c

rMS::pMV261 and rMS::pMV261-*Rv0927c* were shaken at 180 rpm and 37°C in 7H9 medium with a starting absorbance (OD_600_) of 0.02. The OD_600_ of the culture was continuously monitored at an interval of 6 h over 60 h growth period. Subcellular localization of the Rv0927c protein was determined by protocols previously described (21). Briefly, the rMS::pMV261-*Rv0927c* strain was subjected to sonication and the cell wall and cytosol fractions were separated by ultra-centrifuged. Cell wall and cytosol fractions were subjected to Western blot with the same amount of protein. GroEL2 protein served as cytosol marker protein of mycobacteria.

### Macrophages Infection by Recombinant *M. smegmatis*

RAW264.7 cells and BMDMs were seeded into 24-well plates at 5×10^5^ cells per well and cultured overnight. Then the cells were infected with recombinant *M*. smegmatis strains at a multiplicity of infection (MOI) of 10 or with *M. smegmatis* (MOI 1/10) and treated with recombinant Rv0927c protein (5 μg/ml). After 2 h, cells were washed three times with PBS and incubated again with the DMEM medium supplemented with 10 μg/μl gentamicin to kill the bacteria outside macrophages. After 12, 24 and 48 h infection, cells were lysed by 0.05% Triton X-100 for intracellular bacterial counting. The cell lysates were serially ten-fold diluted and then plated on 7H10 agar plates and the colonies were numerated after 3 days.

### Assay for Cytokines Production

After infection for 6, 24 and 48 h, cell culture media were collected for quantitative determination of the lactate dehydrogenase (LDH) activities by LDH Cytotoxicity Assay Kit (Beyotime, Haimen, China) and the proinflammatory cytokines IL-6, TNF-α and IL-1β by employing BD OptEIA™ Mouse IL-6 ELISA Kit (BD), BD OptEIA™ Mouse TNF ELISA Kit (BD) and Mouse IL-1 beta/IL-1F2 DuoSet ELISA (R&D Systems, Minneapolis, MN, USA) according to the manufacturer’s manual. Total mRNA from infected mice tissues was extracted using the RNeasy Plus Mini kit (QIAGEN, Hilden, Germany), followed by reverse transcription. mRNA levels of proinflammatory cytokines IL-6, TNF-α and IL-1β were determined by RT-PCR using primers listed in **Table 1**.

### Luciferase Assay

We transfected RAW264.7 cells with pCMV-*Myc*, pCMV-*Rv0927c* and NF-κB or AP-1 reporter plasmid (250 ng), as well as the control plasmid pRL-TK (50 ng) by using Lipofectamin^®^ 3000. Cells were harvested to determine the luciferase activities by Dual-Luciferase Reporter Assay System (Promega, Madison, WI, USA), following infection with *M. smegmatis*. Results were normalized by pRL-TK-derived Renilla luminescence.

### Subcellular Localization of p65

HEK293-TLR4 cells (InvivoGen, San Diego, CA, USA) were transfected with pCMV-*Myc* or pCMV-*Rv0927c*. Following transfection for 24 h, cells were stimulated by LPS (100 ng/ml) for 15 min. In the protein assay, HEK293-TLR4 cells were treated with Rv0927c (5 μg/ml) for 2 h followed by LPS for 15 min. Then p65 was stained using primary rabbit anti-p65 antibody (Abcam, Cambridge, MA, USA) and secondary Alexa 488-conjugated antibody (Abcam), while nuclei were stained using DAPI (BD). All samples were observed using Leica TCS SP8 STED fluorescence microscope (Leica Microsystems, Wetzlar, Germany).

### Immunoblotting Analysis

Macrophages were infected with recombinant *M. smegmatis* strains at MOI of 10 for indicated times and lysed in Western blot sample buffer. Cell lysates were loaded onto SDS-PAGE and the separated protein were transferred to a polyvinyldifluoride membrane. Membranes were blocked with 5% skim milk for 1 h at room temperature, followed by overnight incubation with primary antibody at 4°C. Next day, the membranes were incubated at room temperature for 1 h with HRP-conjugated secondary antibodies. Images were visualized with ECL chemiluminescence substrate (Thermo Scientific, Waltham, MA, USA) using Amersham Imager 600 Imaging System (GE Healthcare Life Sciences, Pittsburgh, PA, USA). The intensity of the bands was analyzed by Image-J and normalized by β-actin.

The primary antibodies used in this study were as follows: anti-Phospho-IκBα antibody (2859S, Cell Signaling Technology, Danvers, MA, USA), anti-p65 antibody (ab16502, Abcam), anti-Phospho-p65 antibody (3033S, Cell Signaling Technology), anti-SAPK/Jnk antibody (9252S), anti-Phospho-SAPK/Jnk antibody (4671S, Cell Signaling Technology), anti-p44/42 MAPK (ERK1/2) antibody (9102S, Cell Signaling Technology), anti-Phospho-p44/42 MAPK (ERK1/2) antibody (4377S, Cell Signaling Technology), anti-p38 MAPK antibody (8690S, Cell Signaling Technology), anti-Phospho-p38 MAPK antibody (9215S, Cell Signaling Technology), anti-Phospho-TAK1 antibody (9339S, Cell Signaling Technology), anti-TAK1 antibody (5206S, Cell Signaling Technology), anti-IRAK1 antibody (4504S, Cell Signaling Technology) and anti-β-actin antibody (A5441, Sigma-Aldrich, St. Louis, MO, USA). The secondary antibodies were goat anti-mouse IgG-HRP (401215, Sigma-Aldrich) and goat anti-rabbit IgG-HRP (ab6721, Abcam).

### Statistical Analysis

Prism V6.01 software (GraphPad; GraphPad Software, San Diego, USA) was adopted for statistical analyses. Data were shown as mean ± SEM and a two-tailed unpaired *t*-test was used to assess differences between groups. Statistical significance was determined at *p* values of < 0.05 (*), < 0.01 (**).

## Results

### The Expression of Rv0927c in Recombinant *M. smegmatis*

**Figure 1:**
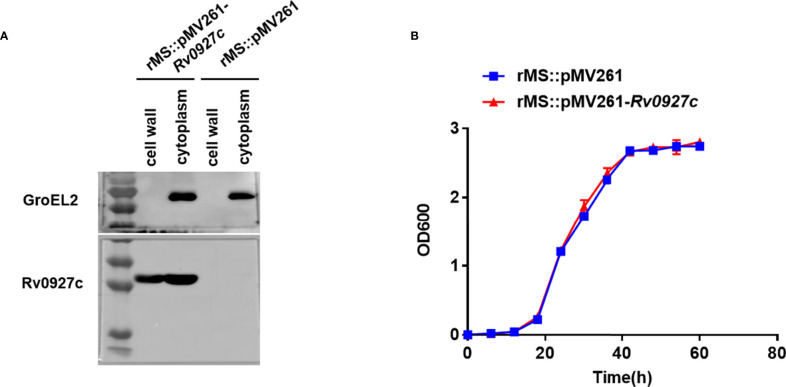
Subcellular localization and bacterial growth. **(A)** Cell fractionations were prepared and subjected to Western blot to detect the subcellular localization of Rv0927c in rMS::pMV261-*Rv0927c*. The cytoplasm marker of *Mycobacterium tuberculosis* GroEL2 served as a control. **(B)** Growth curves were determined for rMS::pMV261 and rMS::pMV261-*Rv0927c* cultivated in 7H9 media.

The *M. tuberculosis* gene *Rv0927c* encodes a 27 kDa protein. This study, we successfully constructed an Rv0927c-overexpressing *M. smegmatis* strain (rMS::pMV261-*Rv0927c*) (**Supplementary Figures 1A, B**) and express rHis-Rv0927c protein (**Supplementary Figure 1C**). rMS::pMV261-*Rv0927c* cells were lysed and their protein fractions were separated for immunoblot analysis of Rv0927c protein expression. We found that Rv0927c was associated with cytosolic and cell wall fractions (**Figure 1A**), corresponding to the previous report (22).

To determine the effect of Rv0927c on the *in vitro* growth of *M. smegmatis*, the growth rates of a strain with empty vector (rMS::pMV261) and rMS::pMV261-*Rv0927c* were monitored. We found no significant difference in growth kinetics between the two recombinant strains (**Figure 1B**); thus, Rv0927c did not influence the growth of *M. smegmatis*.

### Rv0927c Inhibits Inflammatory Cytokine Expression in Macrophages

**Figure 2:**
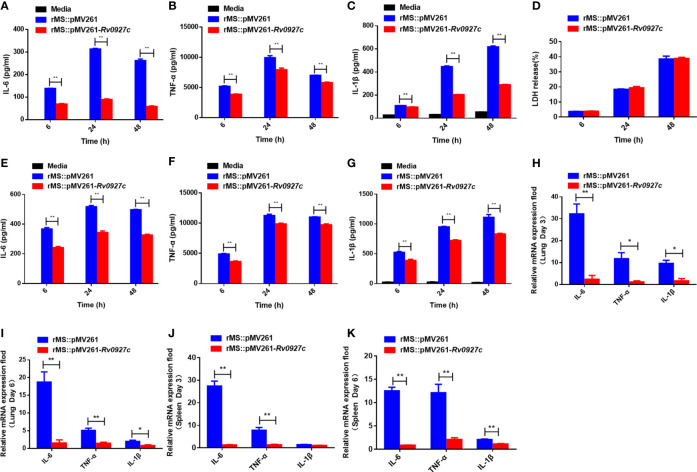
Rv0927c inhibits host inflammatory responses. **(A–C)** RAW264.7 cells and (E-G) BMDMs (5×10^5^ cells/well) were infected with rMS::pMV261 and rMS::pMV261-*Rv0927c*. At the indicated time, culture supernatants were collected and the production of IL-6 **(A, E)**, TNF-α **(B, F)**, and IL-1β **(C, G)** was determined by ELISA. **(D)** The release of LDH was also measured with the culture supernatants. **(H–K)** Quantitative PCR analysis of IL-6, TNF-α, and IL-1β mRNA in the lung and spleen cells from mice infected with rMS::pMV261 or rMS::pMV261-*Rv0927c* strain. *P < 0.05 and **P < 0.01 (unpaired two-tailed Student’s *t* test). Data are representative of experiments with at least three independent biological replicates (mean and sem of *n* = 3 cultures).

To explore the influence of Rv0927c on host immunity, we investigated whether Rv0927c regulates inflammatory cytokines expression in macrophages. RAW264.7 cells (**Figures 2A–C**) and BMDMs (**Figures 2E–G**) infected with rMS::pMV261-*Rv0927c* induced a decrease in IL-6, TNF-α, and IL-1β expression, compared with those infected with rMS::pMV261 at 6, 24, and 48 h post-infection. Cell death was not responsible for this effect (**Figure 2D**). Consistent with this finding, Rv0927c protein can inhibit the expression of the proinflammatory cytokines IL-6, TNF-α, and IL-1β in *M. smegmatis-*infected RAW264.7 cells (**Supplementary Figures 2A–C**) or BMDMs (**Supplementary Figures 2E–G**). Furthermore, we found that overexpression of Rv0927c in *M. smegmatis* decreased the mRNA levels of IL-6, TNF-α, and IL-1β in the lungs and spleens of infected mice (**Figures 2H–K**). These results further demonstrate that Rv0927c inhibits the inflammatory response triggered by *M. smegmatis*.

### Inhibition of Inflammatory Cytokines Expression by Rv0927c Is Mediated by p38 and NF-κB Signaling

**Figure 3:**
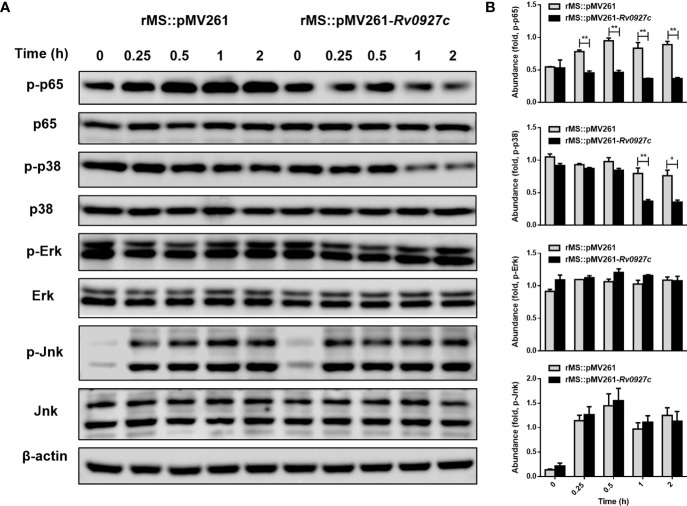
Rv0927c suppresses the activation of MAPK and NF-κB pathways upon mycobacterial infection. **(A)** Immunoblot analysis of phosphorylated p65, p38, Jnk, Erk, and total β-actin in RAW264.7 cells infected for 0–2 h with rMS::pMV261 and rMS::pMV261-*Rv0927c*. **(B)** Densitometry quantification of results for **(A)** presented relative to those of β-actin. *P < 0.05 and **P < 0.01 (unpaired two-tailed Student’s *t* test). Data are representative of one experiment with two independent biological replicates (mean and sem of *n* = 3 cultures).

Infection by *M. tuberculosis* is known to induce inflammatory cytokines by activating the NF-κB and MAPK pathways. To explore the effect of Rv0927c on these signaling pathways, RAW264.7 cells were infected with rMS::pMV261 or rMS::pMV261-*Rv0927c* for 0−2 h. Subsequently, we determined the phosphorylation levels of p65, Erk, p38, and Jnk in the cells by Western blot. The results showed that rMS::pMV261-*Rv0927c* induced a decrease in p65 and p38 phosphorylation (**Figure 3**) but did not affect Jnk and Erk pathways compared with the control strain. Similarly, Rv0927c protein reduced the levels of p65 and p38 phosphorylation in *M. smegmatis*-infected macrophages (**Supplementary Figure 3A**).

**Figure 4:**
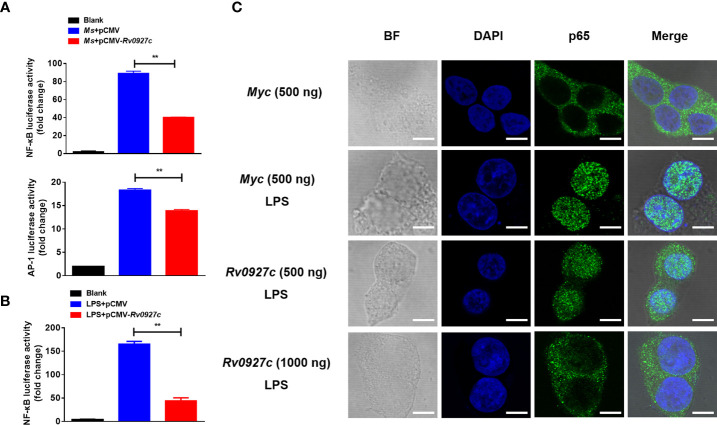
Rv0927c suppresses luciferase activities of NF-κB and AP-1 and inhibits the LPS-induced nuclear translocation of p65. **(A)** RAW264.7 cells (2×10^5^ cells/well) were transfected with NF-κB or AP-1 reporter plasmid (250 ng) and pRL-TK control (10 ng), as well as plasmids encoding *Myc* (250 ng) or *Rv0927c* (250 ng). After infection with *M. smegmatis*, cells were collected to determine the luciferase activities by Dual-Luciferase^®^ Reporter Assay System. **(B)** HEK293-TLR4 cells (2×10^5^ cells/well) were transfected with pCMV-*Myc* (250 ng) or pCMV-*Rv0927c* (250 ng) and NF-κB promoter luciferase reporter constructs (250 ng), as well as plasmid pRL-TK (10 ng). After stimulation with LPS (100 ng/ml) for 5 h, cells were harvested to measure the luminescence. **(C)** HEK293-TLR4 cells were transfected with *Rv0927c* or empty vector. The translocation of p65 was observed using a fluorescence microscope after LPS stimulation for 0.25 h Scale bars, 5 μm. **P < 0.01 (unpaired two-tailed Student’s *t* test). Data are representative of experiments with at least three independent biological replicates (mean and sem of *n* = 3 cultures).

Furthermore, we explored the effect of Rv0927c on NF-κB and AP-1 (a downstream transcription factor of the MAPK pathway) luciferase activity in macrophages stimulated by *M. smegmatis*. The results showed that Rv0927c markedly inhibited the level of NF-κB luciferase activity induced by *M. smegmatis*, while Rv0927c slightly inhibited the level of AP-1 luciferase activity (**Figure 4A**). Rv0927c also greatly inhibited the level of NF-κB luciferase activities in RAW264.7 cells stimulated with several TLRs ligands (**Supplementary Figure 3C**) or HEK293-TLR4 cells stimulated by LPS (**Figure 4B**). The amino acid residues 1−30 of Rv0927c were the most effective in attenuating NF-κB activation (**Supplementary Figures 4A, B**). Next, we examined the translocation of NF-κB p65 in HEK293-TLR4 cells by confocal microscopy. After LPS-induced NF-κB activation, subunit p65 translocated into the nucleus of HEK293 cells. However, overexpression of Rv0927c (**Figure 4C**) or addition of Rv0927c protein (**Supplementary Figure 4C**) could inhibit the translocation of p65.

**Figure 5:**
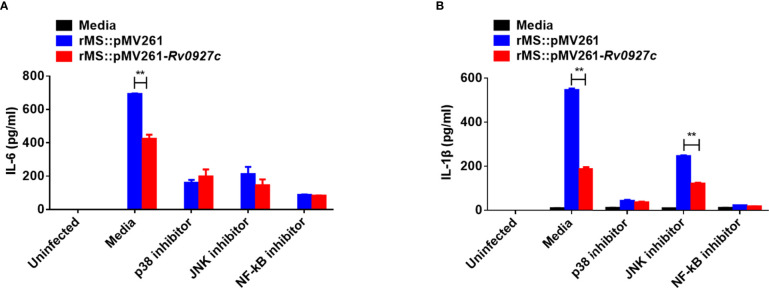
Inhibition of proinflammatory cytokines by Rv0927c requires the NF-κB and p38 pathways. RAW264.7 cells were pretreated with the p38, Jnk, or NF-κB inhibitor for 1 h and then infected rMS::pMV261 and rMS::pMV261-*Rv0927c* for 24 h (MOI = 10). Production of IL-6 **(A)** and IL-1β **(B)** in the culture supernatants was determined by ELISA. **P < 0.01 (unpaired two-tailed Student’s *t* test). Data are representative of one experiment with two independent biological replicates (mean and sem of *n* = 3 cultures).

Then, we investigated the possible involvement of these signaling pathways in Rv0927c inhibition of proinflammatory responses by ELISA. The p38 or NF-κB block attenuated the reduced levels of IL-6 and IL-1β expression in macrophages infected with rMS::pMV261-*Rv0927c* (**Figures 5A, B**), suggesting that Rv0927c inhibited the *M. smegmatis*-induced expression of proinflammatory cytokines through the NF-κB and p38 pathways.

### Rv0927c Suppresses Mycobacteria-Induced NF-κB Pathways Initiation by Downregulating the Phosphorylation Level of IκBα

**Figure 6:**
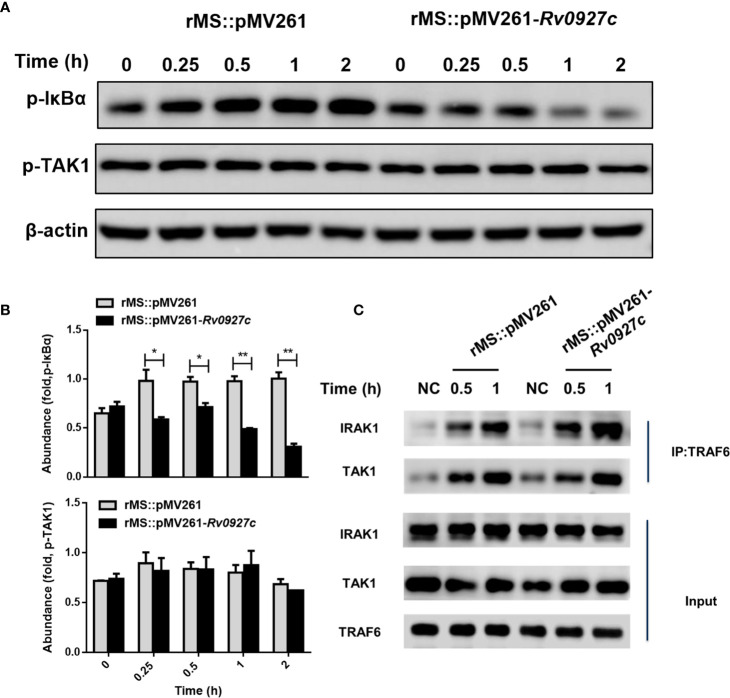
Rv0927c attenuates *M. smegmatis*-induced activation of NF-κB by suppressing the phosphorylation of IκBα. **(A)** Immunoblot analysis of phosphorylated IκBα and TAK1 and total β-actin in RAW264.7 cells infected for 0–2 h with rMS::pMV261 and rMS::pMV261-*Rv0927c*. **(B)** Densitometry quantification of results for **(A)** presented relative to those of β-actin. **(C)** Immunoblot analysis of proteins immunoprecipitated with anti-TRAF6 from lysates of RAW264.7 cells infected with recombinant *M. smegmatis* strains. *P < 0.05 and **P < 0.01 (unpaired two-tailed Student’s *t* test). Data are representative of experiments with at least three independent biological replicates (mean and sem of *n* = 3 cultures).

Activation of TLR signaling pathways promotes the binding of MyD88 molecules to IRAK4 and IRAK1. The complexes are recruited to TRAF6 and TAK1, resulting in the phosphorylation of IκBα, followed by the ubiquitination of IκBα and release of NF-κB (23, 24). Previously, we observed that the phosphorylation and translocation of p65 were inhibited by Rv0927c. To identify the target of Rv0927c inhibition of NF-κB, we explored whether the phosphorylation of TAK1 and IκBα were altered by Rv0927c. The results showed that Rv0927c inhibited the phosphorylation of IκBα but not TAK1 (**Figures 6A, B** and **Supplementary Figure 3B**). Further, we determined whether Rv0927c affected signalosome formation located upstream of IKK activation. RAW264.7 cells were infected with recombinant *M. smegmatis* or remained untreated and cellular proteins were extracted at different times. Endogenous TAK1 and IRAK1 were immunoprecipitated from RAW264.7 cells by TRAF6. TRAF6 was found to interact with IRAK1 and TAK1 after infection with different strains, but the binding of TRAF6 to IRAK1 or TAK1 was not different in RAW264.7 cells infected with rMS::pMV261 or rMS::pMV261-*Rv0927c* (**Figure 6C**). These results suggest that Rv0927c did not affect signalosome formation but did inhibit the activation of NF-κB by downregulating IκBα phosphorylation.

### Rv0927c Enhances *M. smegmatis* Survival in Macrophages and Mice

**Figure 7:**
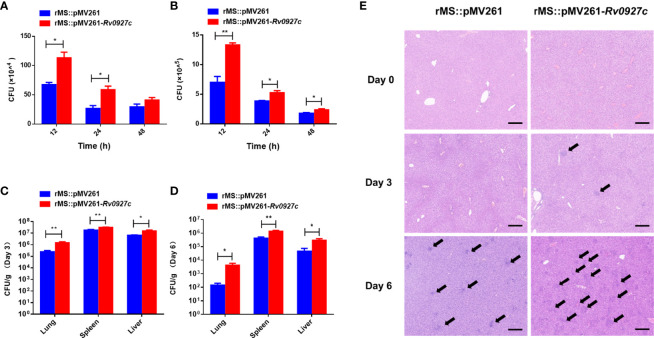
Rv0927c enhances *M. smegmatis* survival and liver pathology of infected mice. **(A, B)** RAW264.7 cells and BMDMs were infected with rMS::pMV261 and rMS::pMV261-*Rv0927c* (MOI=10). Cells were lysed at the indicated time by 0.05% Triton X-100 and the bacterial number was determined by 7H10 plate count. **(C, D)** C57BL/6 mice were given 5×10^7^ CFU of recombinant *M. smegmatis* by intraperitoneal injection. On day 3 or 6 post-infection, mice were sacrificed and the lungs, spleens, and livers were collected for analysis of CFUs. **(E)** Hematoxylin and eosin (H&E)**** staining of the livers of mice treated as in **(C, D)**; arrows indicate foci of cellular infiltration. Scale bars, 200 μm. *P < 0.05 and **P < 0.01 (unpaired two-tailed Student’s *t* test). Data are representative of one experiment with two independent biological replicates (mean and sem of *n* = 5 mice per group).

To determine whether Rv0927c could promote mycobacterial survival in macrophages, intracellular bacteria in the RAW264.7 cells and BMDMs infected with rMS::pMV261 or rMS::pMV261-*Rv0927c* were harvested at 12, 24, and 48 h post-infection and the colony-forming units (CFUs) were determined. The bacterial load of macrophages infected with rMS::pMV261-*Rv0927c* was higher than that of macrophages infected with rMS::pMV261 (**Figures 7A, B**). Consistent with this finding, Rv0927c protein was also found to enhance the survival of *M. smegmatis* in macrophages (**Supplementary Figures 2D, H**). Further, overexpression of Rv0927c in *M. smegmatis* leads to increased bacterial burden in the spleens, livers, and lungs of mice (**Figures 7C, D**).

The induction of cellular immunity mediated by T_H_1 cells is essential to defend against intracellular pathogens. Immune cell infiltration indicates a failure of cellular immune in response to mycobacteria (16, 25). Small foci of cellular infiltration began to appear on day 3 and became prominent on day 6 in the livers of the mice. The liver tissues of C57BL/6 mice infected with rMS::pMV261-*Rv0927c* showed more immune cell infiltration than those infected with rMS::pMV261 (**Figure 7E**). These results indicate that Rv0927c may be involved in the pathogenesis of mycobacterial disease.

## Discussion

*M. tuberculosis* is mainly transmitted through the air (26). It enters the lungs from the respiratory tract and infects macrophages. These macrophages are the main initial effector cells to resist *M. tuberculosis* infection (27). Infected macrophages secrete proinflammatory cytokines, which play a crucial role in the inflammatory response and the outcome of mycobacterial infections (28, 29). However, *M. tuberculosis* can suppress the expression of proinflammatory cytokines to avoid immune surveillance (30–32). Thus, the factors that suppress the expression of these proinflammatory cytokines are the ideal targets to control *M. tuberculosis* infection. *M. tuberculosis* Rv0927c has been reported as a target for identifying *M. tuberculosis* W-Beijing strains (18, 33–36) and is known to alter the secretion of cytokines (19). Here, Rv0927c was found to inhibit the expression of IL-6, TNF-α, and IL-1β in RAW264.7 cells, and the transcription of IL-6, TNF-α, and IL-1β in mouse tissues. To eliminate interference from other factors (15), we also determined the growth rate of rMS::pMV261-*Rv0927c* and its ability to induce cell death. The results showed that overexpression of Rv0927c did not change these characteristics. It has been reported that IL-6 or TNF-α knockout mice display increased susceptibility to mycobacteria (37, 38). Like TNF-α, IL-1β is also a key proinflammatory cytokine involved in the host response to *M. tuberculosis*. IL-1R type I-deficient mice display increased mycobacterial outgrowth and defective granuloma formation after infection with *M. tuberculosis* (39). Correspondingly, Rv0927c was found to enhance mycobacterial survival *in vivo* and *in vitro*.

Cells can respond to extracellular signals *via* NF-κB and MAPK signaling pathways, which regulate the production of proinflammatory cytokines by macrophages infected with *M. tuberculosis* (40, 41). We examined the activation status of the signaling molecules involved in these pathways. rMS::pMV261-*Rv0927c* induced a decrease in p65 and p38 phosphorylation, compared with the control strains. Luciferase and immunofluorescence assays showed that the activation of NF-κB and AP-1 was suppressed by Rv0927c. Wang et al. demonstrated that inhibition of p38, Jnk, or NF-κB attenuated the enhanced expression of IL-1β and IL-6 in macrophages infected with H37RvΔRv0222 (15). In this study, the NF-κB and p38 pathways were found to be involved in Rv0927c-induced IL-6 and IL-1β expression, suggesting that Rv0927c suppresses *M. smegmatis*-induced expression of proinflammatory cytokines by downregulating the activation of NF-κB and p38 pathways. Compared with MAPK, Rv0927c inhibits NF-κB activation to a greater extent. Thus, we next explored the molecular mechanism of Rv0927c regulating NF-κB pathway. Of course, the p38 pathway is also important and related work still remains to be continued.

TLRs are the primary sensors in recognizing microbial components and induce host immune responses (42). The main signaling pathways of TLRs activate the adaptor molecule TRAF6, leading to the phosphorylation of TAK1 and culminating in the activation of MAPK and NF-κB to the biosynthesis of proinflammatory cytokines (43). It has been reported that inactivation of TAK1 prevents the activation of NF-κB, Jnk, and p38 pathways (44). In this study, we found that Rv0927c inhibited the phosphorylation of p65 and p38 but not that of Jnk, ERK, and TAK1. In addition, Rv0927c did not affect IRAK1, TRAF6, or TAK1 signalosome formation, suggesting that TAK1 may not be a target for Rv0927c to suppress NF-κB and p38 pathways. Activated TAK1 will trigger IκBα phosphorylation (45) and the phosphorylation level of IκBα was found to be inhibited by Rv0927c. However, the mechanism as to how Rv0927c regulates IκBα phosphorylation requires further study.

In summary, we provide evidence that *M. tuberculosis* Rv0927c suppresses host proinflammatory cytokine secretion and enhances mycobacterial survival with the additional consequence of host pathology. Our findings identify a novel *M. tuberculosis* pathogenic factor that modulates host immunity, providing a potential drug target for therapeutic intervention in TB.

## Data Availability Statement

The raw data supporting the conclusions of this article will be made available by the authors, without undue reservation.

## Ethics Statement

The animal study was reviewed and approved by Animal Welfare and Ethics Committees of Yangzhou University.

## Author Contributions

ZX, XJ, and AX designed and coordinated the study. AX, XL, and JQ performed the experiments, and AX and ZX wrote the manuscript. AX, XL, ZX, and XC analyzed the data. ZX and XJ reviewed and edited the manuscript. All authors contributed to the article and approved the submitted version.

## Funding

This work was supported by the Science and Technology Program of Jiangsu (BK20201432), the Science and Technology Innovation Cultivation Program of Yangzhou University (2019CXJ158), the independent program of Jiangsu Key Laboratory of Zoonosis (RZZ202003) and the Qinglan Project and Priority Academic Development Program of Jiangsu Higher Education Institutions (PADP).

## Conflict of Interest

The authors declare that the research was conducted in the absence of any commercial or financial relationships that could be construed as a potential conflict of interest.

## Publisher’s Note

All claims expressed in this article are solely those of the authors and do not necessarily represent those of their affiliated organizations, or those of the publisher, the editors and the reviewers. Any product that may be evaluated in this article, or claim that may be made by its manufacturer, is not guaranteed or endorsed by the publisher.

## Supplementary Material

Supplementary Figure 1 Construction of recombinant *M. smegmatis* overexpressing Rv0927c and preparation of Rv0927c protein. **(A)** Rv0927c was amplified with a 792 bp DNA fragment. **(B)** Expression of His-tagged Rv0927c protein in rMs::pMV261-*Rv0927c* was determined by Western blot. Lane 1−2: whole cell lysate of rMs::pMV261; Lane 3−4: whole cell lysate of rMs::pMV261-*Rv0927c*. **(C)** SDS-PAGE analysis of rHis-Rv0927c protein. Lane 1: purified recombinant Rv0927c (27 kDa).

Supplementary Figure 2 Effect of Rv0927c on proinflammatory cytokines production of *M. smegmatis*-infected macrophages and the survival of *M. smegmatis* in macrophages. **(A–C)** RAW264.7 cells and **(E–G)** BMDMs were infected with *M. smegmatis* (MOI= 10) together with the recombinant Rv0927c protein (5 μg/ml) for 6, 24, 48 h. Culture supernatants were collected and the production of IL-6 **(A, E)**, TNF-α **(B, F)**, and IL-1β **(C, G)** was examined using ELISA assay. **(D)** RAW264.7 cells and (H) BMDMs were infected with *M. smegmatis* (MOI= 10) with or without the Rv0927c protein (5 μg/ml) treatment for 12, 24, 48 h. A CFU assay was performed to assay *M. smegmatis* survival. **P* < 0.05 and ***P* < 0.01 (unpaired two-tailed Student’s *t* test). Data are representative of one experiment with two independent biological replicates (mean and sem of n = 3 cultures).

Supplementary Figure 3 Effect of Rv0927c on p65, p38, Erk, jnk, IκBα and TAK1 phosphorylation levels and NF-κB luciferase activity in macrophages stimulated with several TLR ligands. **(A, B)** RAW264.7 cells were infected with *M. smegmatis* (MOI= 10) together with the recombinant Rv0927c protein (5 μg/ml) for the indicated time. Total protein was extracted and examined by immunoblot analysis. **(B)** RAW264.7 cells were transfected with Rv0927c or empty vector, as well as NF-κB reporter plasmid plus pRL-TK. Cells were stimulated with ligands for TLR2 (PGN), TLR1/2 (Pam3CSK4), TLR3 (Poly(I:C)), TLR4 (LPS), TLR7/8 (R848), and TLR9 (ODN1826), following 24 h transfection. Luciferase activity in cellular extracts was measured. ***P* < 0.01 (unpaired two-tailed Student’s *t* test). Data are representative of experiments with at least three independent biological replicates (mean and sem of n = 3 cultures).

Supplementary Figure 4 Effect of 30 amino acids at the N-terminus of Rv0927c on NF-κB activation and effect of Rv0927c on LPS-induced nuclear translocation of p65. **(A)** Nucleic acid sequences encoding amino acids 31−263 (Lane 1), 61−263 (Lane 2), 91−263 (Lane 3), 121−263 (Lane 4), 151−263 (Lane 5), 1−233 (Lane 6), 1−203 (Lane 7), 1−173 (Lane 8), 1−143 (Lane 9), and 1−113 (Lane 10) of Rv0927c were amplified with gene-specific primers listed in Supplementary Table 1 and cloned into pCMV-Myc. **(B)** HEK293-TLR4 cells were transfected with 10 eukaryotic plasmid deletion mutants of Rv0927c, as well as plasmid NF-κB reporter and pRL-TK. After stimulation with LPS, cells were harvested to measure the luminescence. **(C)** HKE293-TLR4 cells were pre-incubated with Rv0927c (5 μg/ml) for 2 h and then were treated with LPS for 0.25 h. The translocation of p65 was observed using fluorescence microscope. Scale bars, 5 μm. **P* < 0.05 and ***P* < 0.01 (unpaired two-tailed Student’s *t* test). Data are representative of one experiment with two independent biological replicates (mean and sem of n = 3 cultures).
