# Supplementary figures and images for: Mycobacterium tuberculosis Rv0927c Inhibits NF-κB Pathway by Downregulating the Phosphorylation Level of IκBα and Enhances Mycobacterial Survival
Source: Front Immunol. 2021 Aug 31;12:721370. doi: 10.3389/fimmu.2021.721370 (PMC8438533; doi:10.3389/fimmu.2021.721370)

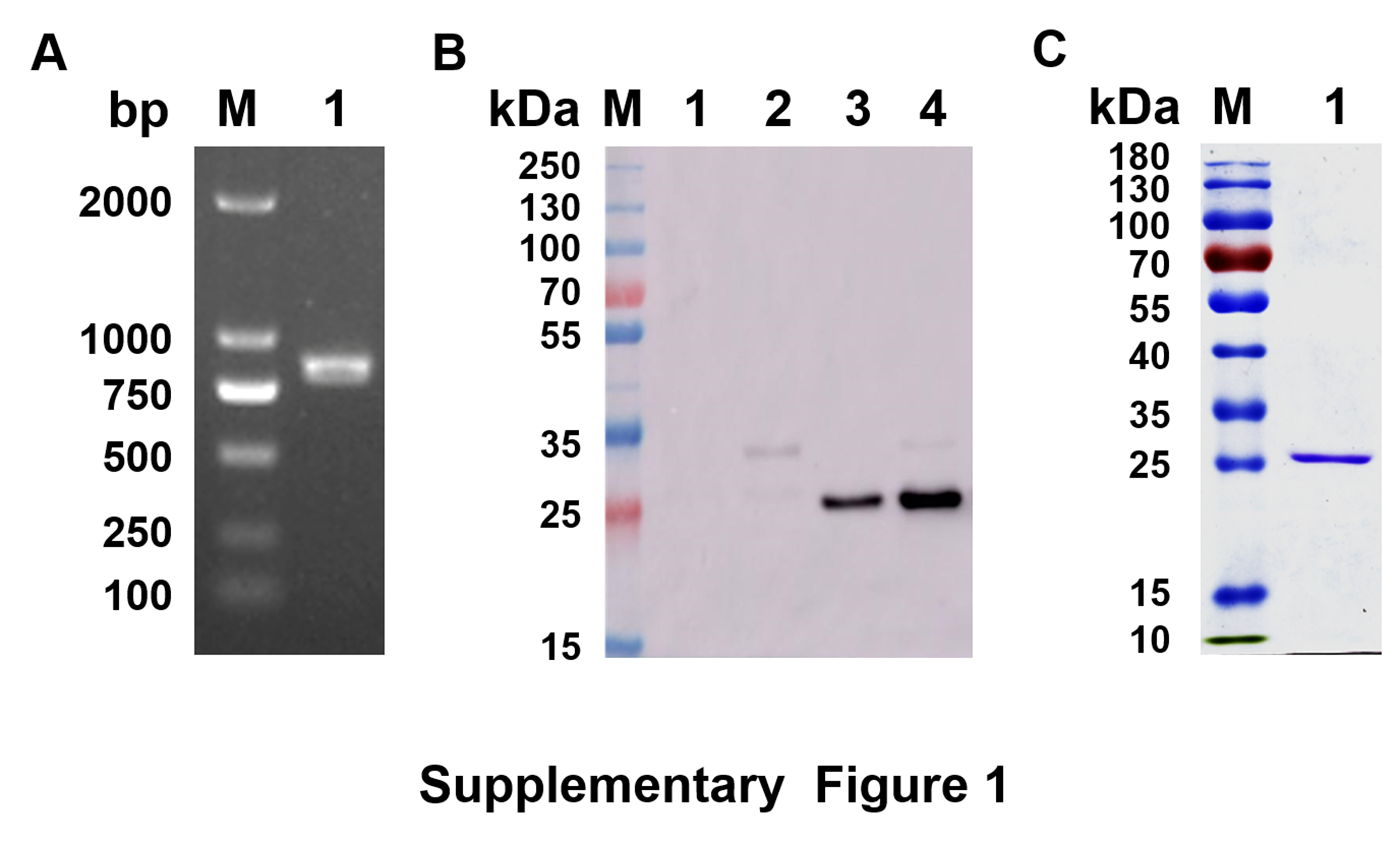

Supplement: Supplementary Figure 1 — Construction of recombinant M. smegmatis overexpressing Rv0927c and preparation of Rv0927c protein. (A) Rv0927c was amplified with a 792 bp DNA fragment. (B) Expression of His-tagged Rv0927c protein in rMs::pMV261-Rv0927c was determined by Western blot. Lane 1−2: whole cell lysate of rMs::pMV261; Lane 3−4: whole cell lysate of rMs::pMV261-Rv0927c. (C) SDS-PAGE analysis of rHis-Rv0927c protein. Lane 1: purified recombinant Rv0927c (27 kDa). [file Image_1.tif]

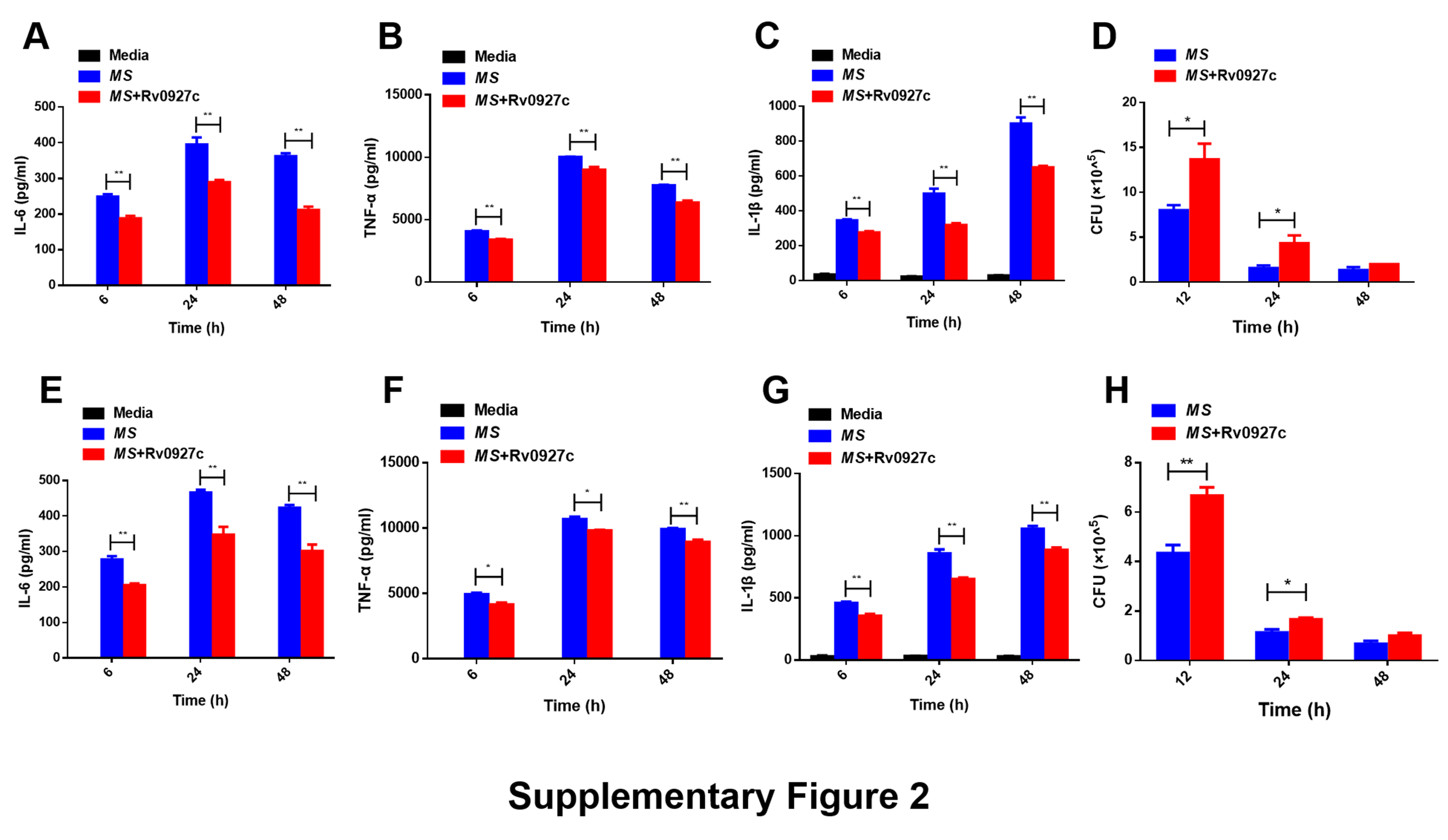

Supplement: Supplementary Figure 2 — Effect of Rv0927c on proinflammatory cytokines production of M. smegmatis-infected macrophages and the survival of M. smegmatis in macrophages. (A–C) RAW264.7 cells and (E–G) BMDMs were infected with M. smegmatis (MOI= 10) together with the recombinant Rv0927c protein (5 μg/ml) for 6, 24, 48 h. Culture supernatants were collected and the production of IL-6 (A, E), TNF-α (B, F), and IL-1β (C, G) was examined using ELISA assay. (D) RAW264.7 cells and (H) BMDMs were infected with M. smegmatis (MOI= 10) with or without the Rv0927c protein (5 μg/ml) treatment for 12, 24, 48 h. A CFU assay was performed to assay M. smegmatis survival. *P < 0.05 and **P < 0.01 (unpaired two-tailed Student’s t test). Data are representative of one experiment with two independent biological replicates (mean and sem of n = 3 cultures). [file Image_2.tif]

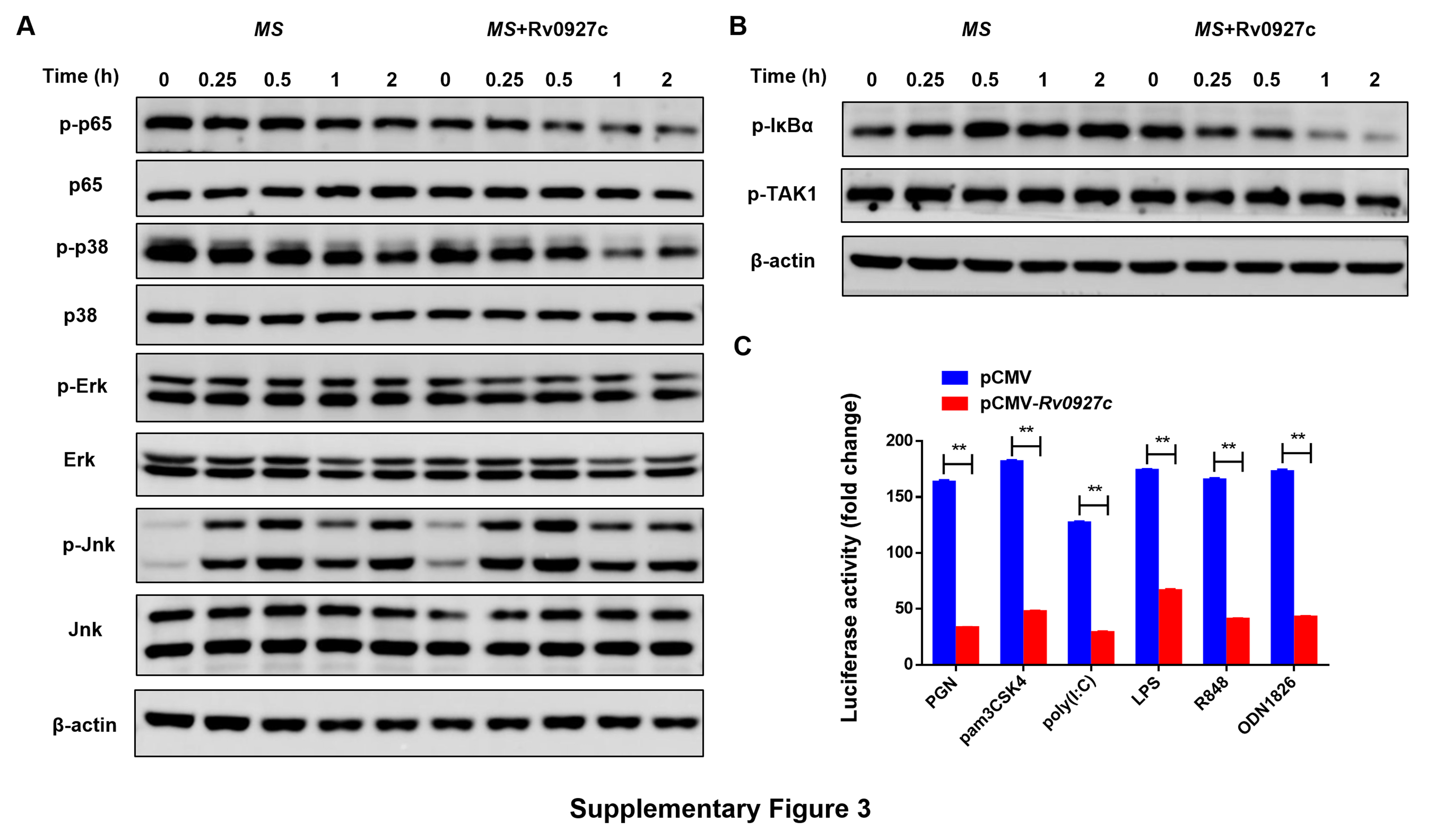

Supplement: Supplementary Figure 3 — Effect of Rv0927c on p65, p38, Erk, jnk, IκBα and TAK1 phosphorylation levels and NF-κB luciferase activity in macrophages stimulated with several TLR ligands. (A, B) RAW264.7 cells were infected with M. smegmatis (MOI= 10) together with the recombinant Rv0927c protein (5 μg/ml) for the indicated time. Total protein was extracted and examined by immunoblot analysis. (B) RAW264.7 cells were transfected with Rv0927c or empty vector, as well as NF-κB reporter plasmid plus pRL-TK. Cells were stimulated with ligands for TLR2 (PGN), TLR1/2 (Pam3CSK4), TLR3 (Poly(I:C)), TLR4 (LPS), TLR7/8 (R848), and TLR9 (ODN1826), following 24 h transfection. Luciferase activity in cellular extracts was measured. **P < 0.01 (unpaired two-tailed Student’s t test). Data are representative of experiments with at least three independent biological replicates (mean and sem of n = 3 cultures). [file Image_3.tif]

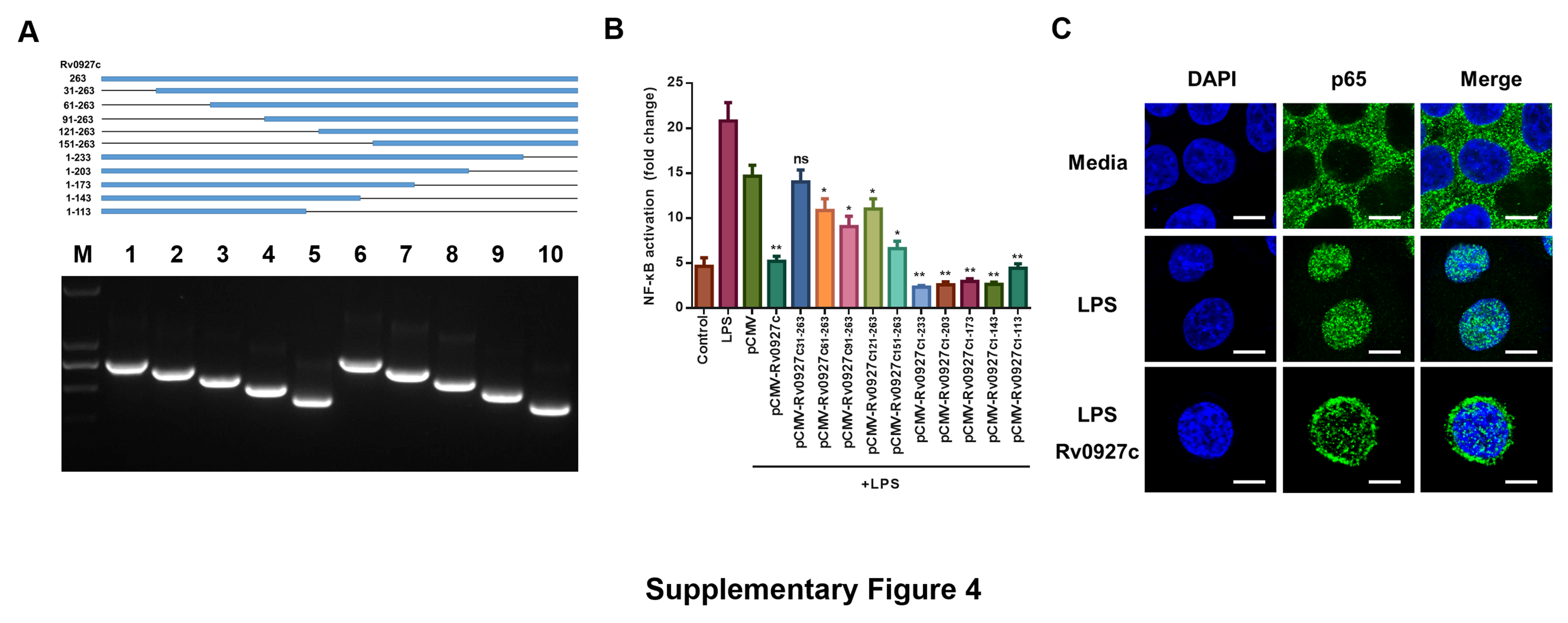

Supplement: Supplementary Figure 4 — Effect of 30 amino acids at the N-terminus of Rv0927c on NF-κB activation and effect of Rv0927c on LPS-induced nuclear translocation of p65. (A) Nucleic acid sequences encoding amino acids 31−263 (Lane 1), 61−263 (Lane 2), 91−263 (Lane 3), 121−263 (Lane 4), 151−263 (Lane 5), 1−233 (Lane 6), 1−203 (Lane 7), 1−173 (Lane 8), 1−143 (Lane 9), and 1−113 (Lane 10) of Rv0927c were amplified with gene-specific primers listed in Supplementary Table 1 and cloned into pCMV-Myc. (B) HEK293-TLR4 cells were transfected with 10 eukaryotic plasmid deletion mutants of Rv0927c, as well as plasmid NF-κB reporter and pRL-TK. After stimulation with LPS, cells were harvested to measure the luminescence. (C) HKE293-TLR4 cells were pre-incubated with Rv0927c (5 μg/ml) for 2 h and then were treated with LPS for 0.25 h. The translocation of p65 was observed using fluorescence microscope. Scale bars, 5 μm. *P < 0.05 and **P < 0.01 (unpaired two-tailed Student’s t test). Data are representative of one experiment with two independent biological replicates (mean and sem of n = 3 cultures). [file Image_4.tif]
