# Supplementary material for: Mycobacterium tuberculosis Rv0927c Inhibits NF-κB Pathway by Downregulating the Phosphorylation Level of IκBα and Enhances Mycobacterial Survival
Source: Front Immunol. 2021 Aug 31;12:721370. doi: 10.3389/fimmu.2021.721370 (PMC8438533; doi:10.3389/fimmu.2021.721370)
Supplement: Supplementary file 5 [file Table_1.docx]

**Supplementary Table. 1.** Primers used for construction of Rv0927c deletion mutants

| Primer Name | Primer Sequence |
| --- | --- |
| pCMV-Rv0927c_31-263_-F | TGGCCATGGAGGCCCGAATTCGGGCCCAGGCCGGCGCGGAT (*Eco*R I) |
| pCMV-Rv0927c_31-263_-R | CCGCGGCCGCGGTACCTCGAGTCACAGGTCCGGAATGGGA (*Xho* I) |
| pCMV-Rv0927c_61-263_-F | TGGCCATGGAGGCCCGAATTCGGGCCCACACCGTTGCCGCC (*Eco*R I) |
| pCMV-Rv0927c_61-263_-R | CCGCGGCCGCGGTACCTCGAGTCACAGGTCCGGAATGGGA (*Xho* I) |
| pCMV-Rv0927c_91-263_-F | TGGCCATGGAGGCCCGAATTCGGGTCGTCAACAACGTTGGCG (*Eco*R I) |
| pCMV-Rv0927c_91-263_-R | CCGCGGCCGCGGTACCTCGAGTCACAGGTCCGGAATGGGA (*Xho* I) |
| pCMV-Rv0927c_121-263_-F | TGGCCATGGAGGCCCGAATTCGGACCGCCCACGCGCTGACC (*Eco*R I) |
| pCMV-Rv0927c_121-263_-R | CCGCGGCCGCGGTACCTCGAGTCACAGGTCCGGAATGGGA (*Xho* I) |
| pCMV-Rv0927c_151-263_-F | TGGCCATGGAGGCCCGAATTCGGCGGCTGGCGGCGCGGGGT (*Eco*R I) |
| pCMV-Rv0927c_151-263_-R | CCGCGGCCGCGGTACCTCGAGTCACAGGTCCGGAATGGGA (*Xho* I) |
| pCMV-Rv0927c_1-233_-F | TGGCCATGGAGGCCCGAATTCGGATGATCCTGGATATGTTCCGTCTT (*Eco*R I) |
| pCMV-Rv0927c_1-233_-R | CCGCGGCCGCGGTACCTCGAGTCAGGCCAAATACACTGCCG (*Xho* I) |
| pCMV-Rv0927c_1-203_-F | TGGCCATGGAGGCCCGAATTCGGATGATCCTGGATATGTTCCGTCTT (*Eco*R I) |
| pCMV-Rv0927c_1-203_-R | CCGCGGCCGCGGTACCTCGAGTCAGTTGGCGGCTACCACC (*Xho* I) |
| pCMV-Rv0927c_1-173_-F | TGGCCATGGAGGCCCGAATTCGGATGATCCTGGATATGTTCCGTCTT (*Eco*R I) |
| pCMV-Rv0927c_1-173_-R | CCGCGGCCGCGGTACCTCGAGTCACAGCCGGGTGTAGTGGG (*Xho* I) |
| pCMV-Rv0927c_1-143_-F | TGGCCATGGAGGCCCGAATTCGGATGATCCTGGATATGTTCCGTCTT (*Eco*R I) |
| pCMV-Rv0927c_1-143_-R | CCGCGGCCGCGGTACCTCGAGTCAGATCACGCTGCCGCC (*Xho* I) |
| pCMV-Rv0927c_1-113_-F | TGGCCATGGAGGCCCGAATTCGGATGATCCTGGATATGTTCCGTCTT (*Eco*R I) |
| pCMV-Rv0927c_1-113_-R | CCGCGGCCGCGGTACCTCGAGTCAGTCCGCGAGGTCCTTG (*Xho* I) |
